# Serum adropin levels in psoriasis vulgaris and its relation with metabolic parameters

**DOI:** 10.3906/sag-1712-192

**Published:** 2019-02-11

**Authors:** Selma KORKMAZ, Gülben SAYILAN ÖZGÜN

**Affiliations:** 1 Department of Dermatology, Faculty of Medicine, Süleyman Demirel University, Isparta Turkey; 2 Department of Biochemistry, Faculty of Medicine, Trakya University, Edirne Turkey

**Keywords:** Psoriasis, metabolic syndrome, adropin

## Abstract

**Background/aim:**

Adropin is a peptide-structure hormone that plays a role in preventing the development of insulin resistance, which has been linked to obesity and metabolic regulation. The purpose of this study is to assess serum adropin levels and their relationship with metabolic parameters in psoriasis vulgaris patients both with and without metabolic syndrome (MetS).

**Materials and methods:**

Fifty-three patients and 26 healthy controls were included in this study. Serum adropin levels, fasting blood glucose, fasting serum insulin, high-density lipoprotein cholesterol, low-density lipoprotein cholesterol, total cholesterol, and triglyceride levels of all participants were analyzed. Enzyme-linked immunosorbent assay was used to measure serum adropin levels.

**Results:**

Serum adropin levels were 2.94 ± 0.56 ng/mL in psoriatic patients without MetS, 2.49 ± 0.77 ng/mL in psoriasis patients with MetS, and 3.37 ± 0.71 ng/mL in the control group. Multivariate logistic regression analysis was used to evaluate adropin decreases in psoriasis patients as an independent predictor of the presence of MetS.

**Conclusion:**

The serum levels of adropin in psoriasis patients were significantly lower in the presence of MetS, and this decrease was more prominent than in those without MetS. Adropin may be a contributing factor for metabolic disorders and the development of MetS in psoriasis patients.

## 1. Introduction

Psoriasis vulgaris is a chronic inflammatory disease that is observed in 1%–3% of the general population and is characterized by attack and remission periods (1,2). A combination of insulin resistance and metabolic syndrome (MetS) is commonly observed in psoriasis patients (3–6). These patients also often suffer from hypertension (HT), heart failure, and diabetes mellitus (DM) (7). The mechanism of development of cardiometabolic disorders in psoriasis patients is still not clear.

Adropin is a newly discovered hormone with a peptide structure that plays a role in insulin response and energy metabolism and is released from the liver, brain, and endothelial cells. The expression of adropin is regulated by energy status and dietary nutrient content, and is altered in obesity. Serum adropin levels were reported to decrease in obese patients, females, and the elderly people (8,9). This hormone helps prevent the development of insulin resistance, which has been linked to obesity and metabolic regulation. Studies revealed that in situations of adropin deficiency, glucose homeostasis was disrupted and the amount of body fat increased. Additionally, adropin deficiency was suggested to be a possible risk factor for MetS development and hypertension (6,8,10–12). A reduction in adropin levels may contribute to the development of insulin resistance and dyslipidemia (8). In addition, adropin may be an independent risk factor for endothelial dysfunction in type 2 DM (12).

To the best of our knowledge, serum adropin levels have not yet been assessed in psoriasis patients. Therefore, this study aimed to determine the serum adropin level and its relationship with metabolic parameters in psoriasis patients.

## 2. Materials and methods

The study was initiated following the approval of the local ethics committee. Informed consent was obtained from all participants before the study was commenced.

### 2.1. Patient group and study protocol

The present case-control study was conducted in the Department of Dermatology and enrolled 53 patients with psoriasis. Patients between 18 and 65 years of age who were diagnosed clinically and histopathologically with psoriasis vulgaris were randomly included in the study. The psoriasis patients who consented to participate were then divided into two groups based upon whether they had MetS. MetS was defined according to the modified NCEP/ATP III criteria (13). The healthy control group comprised individuals without any illness or drug use and was randomly selected from volunteers between the ages of 18 and 65. The final study design included 27 patients with psoriasis and MetS (mean age: 45.00 ± 15.00 years; 11 females, 16 males), 26 with psoriasis but without MetS (median age: 41.00 ± 15.00 years; 15 females, 11 males), and 26 healthy controls (median age: 43.00 ± 15.00 years; 10 females, 16 males). The Psoriasis Area and Severity Index (PASI) was used to evaluate erythema, induration, and scaling of the lesions in four body areas (head, trunk, arms, and legs). A PASI score of <10 was assessed as mild, while scores ≥10 were classified as severe and moderate psoriasis (14).

Participants with DM, heart failure, cirrhosis, infection, renal failure, connective tissue diseases, pregnancy, malignancy, endocrinopathies (Cushing’s syndrome, Addison’s disease, polycystic ovarian syndrome, pheochromocytoma, and hyper- or hypothyroidism), current use of hormonal medicines (such as glucocorticoids, statins, oral antidiabetics, and insulin), and severe systemic involvement of psoriasis (such as psoriatic arthritis requiring the use of anti-TNF agents) were excluded from the study. 

The age, weight, height, body mass index (BMI: body weight (kg)/height (cm)2), waist circumference (WC), hip circumference, systolic blood pressure (SBP), and diastolic blood pressure (DBP) of all participants were recorded. Waist and hip circumference lengths were measured and waist-to-hip ratio (WHR) was found. The fasting blood glucose (FBG), fasting serrum insulin, high-density lipoprotein cholesterol (HDL-C), low-density lipoprotein cholesterol (LDL-C), total cholesterol (TC), and triglyceride (TG) levels of all participants were analyzed. 

Fasting serum insulin levels were measured by Centaur XP Immunassay Analyzer using original kits (Siemens Healthcare Diagnostics, Erlangen, Germany). FBG, HDL-C, LDL-C, TC, and TG levels of all participants were measured by Architect C16000 Clinical Chemistry Analyzer using original kits (Abbott Laboratories, Abbott Park, IL, USA). Serum adropin levels were measured by using a commercial enzyme-linked immunosorbent assay (ELISA) kit (Cusobio, Wuhan, China) according to its original method.

Insulin resistance was determined with the homeostasis model assessment (HOMA) calculator.

### 2.2. Statistical analysis

Student’s t-test was used for the comparison of 2 independent groups of normally distributed variables, and the least significant difference (LSD) test was used for paired comparisons to identify the group that contributed to the difference. For nonnormally distributed variables, the Mann–Whitney U test was used to compare 2 independent groups. Dunn’s test was used for post hoc comparisons. Spearman correlation analysis was carried out to identify associations between the parameters in psoriasis patients. Multiple logistic regression analysis was carried out to evaluate the relationship between MetS and adropin and WHR in psoriasis patients. SPSS 15 for Windows was used for all statistical analyses. The level of significance was set at P < 0.05.

## 3. Results

The mean age and sex distributions were similar in both groups (P > 0.05 for each variable). BMI, LDL-C, TG, and FBG levels were significantly higher in the psoriasis group with MetS compared to the psoriasis group without MetS and the healthy control group (P < 0.05 for each parameter) (Table 1).

**Table 1:** Demographic and laboratory characteristics of groups.

Parameters	P (n = 26)	PM (n = 27)	HC (n = 26)	P (P - HC)	P (PM - HC)	P (P - PM)
Age (years)*	41.00 ± 15.00	45.00 ± 15.00	43.00 ± 15.00	0.340	0.795	0.177
Sex (F/M)	15/11	11/16	10/16	0.085	0.772	0.164
BMI (kg/m2)*	27.65 ± 4.12	30.38 ± 7.28	24.73 ± 8.01	0.022	<0.001	0.033
FBG (mg/dL)*	88.00 ± 11.00	104.00 ± 23.00	87.00 ± 5.00	0.438	<0.001	<0.001
TG (mg/dL)	89.00 ± 41.00	198.50 ± 133.00	89.50 ± 53.00	0.227	<0.001	<0.001
LDL-C (mg/dL)	124.00 ± 33.00	139.00 ± 47.00	108.50 ± 33.00	0.138	0.001	0.030
HDL-C (mg/dL)	47.00 ± 10.00	43.00 ± 21.00	54.00 ± 17.00	0.035	0.011	0.345
Insulin (mIU/L)	11.54 ± 12.00	12.15 ± 10.00	9.33 ± 6.00	0.433	0.162	0.701
HOMA-IR	2.76 ± 2.35	3.17 ± 3.59	1.96 ± 1.12	0.165	0.007	0.176
SBP (mmHg)	120.0 ± 13.00	120.00 ± 20.00	115.00 ± 10.00	0.014	0.008	0.389
DBP (mmHg) WHR Hip circumference WC	75.00 ± 10.00 0.93 ± 0.05 105.42 ± 12.83 98.17 ± 14.35	75.00 ± 10.00 0.96 ± 0.06 108.87 ± 10.45 105.22 ± 11.50	70.00 ± 10.00 0.85 ± 0.07 102.6 ± 11.27 87.83 ± 11.27	0.011 0.001 0.417 0.027	0.014 <0.001 0.037 <0.001	0.836 0.82 0.122 0.020
PASI	3.75 ± 7.00	2.10 ± 3.0				0.171
Adropin (ng/mL)	2.94 ± 0.56	2.49 ± 0.77	3.37 ± 0.71	0.024	<0.001	0.024

Data expressed as *mean (±SD) and median (IQR). Psoriasis group, P; psoriasis group with metabolic syndrome, PM; healthy control group, HC; body mass index, BMI; fasting blood glucose, FBG; high-density lipoprotein/low-density lipoprotein cholesterol, HDL-C/ LDL-C; triglyceride, TG; systolic blood pressure, SBP; diastolic blood pressure, DBP; Homeostatic Model Assessment-Insulin Resistance, HOMA-IR; waist-to-hip ratio, WHR; waist circumference, WC; Psoriasis Area Severity Index, PASI.

Compared to the healthy control group, psoriasis patients had significantly lower serum adropin levels (P < 0.05 each) irrespective of whether they had MetS or not. Importantly, psoriasis patients with MetS had significantly lower adropin levels compared to those without MetS (P = 0.024) (Table 1; Figure).

**Figure 1:**
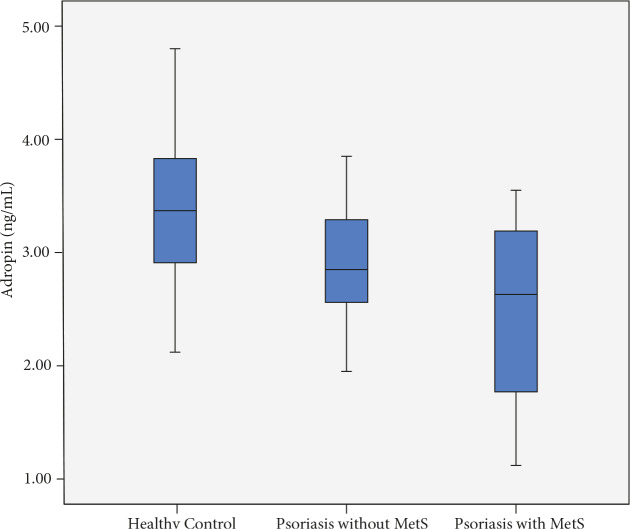
Psoriasis patients both with and without MetS had significantly lower serum adropin levels compared to the healthy control group.

The PASI scores in psoriasis patients both with and without MetS were similar (P > 0.05). A correlation analysis of psoriasis patients revealed a negative correlation between serum adropin levels and WHR, TG, and HOMA-IR (r = –0.338, P = 0.005; r = –0.441, P < 0.001; and r = 0.259, P = 0.027, respectively; Table 2). A multivariate logistic regression analysis identified the reduction in adropin levels in psoriasis patients as an independent predictor for the development of MetS (Table 3). No correlation was identified between the PASI score and adropin levels (r = 0.193, P = 0.188). There was also no correlation between the serum adropin levels and the duration of psoriasis (P = 0.380, r = 0.130). 

**Table 2:** The analysis of the correlation between adropin, metabolic parameters, and PASI in psoriasis patients.

	BMI	WHR	SBP	DBP	HDL-C	TG	FBG	HOMA-IR	PASI
Adropin									
R	–0.107	–0.338	–0.203	–0.133	0.189	–0.441	–0.167	–0.259	0.193
P	0.386	0.005	0.098	0.280	0.114	<0.001	0.164	0.027	0.188
BMI									
R		0.770	0.428	0.460	–0.197	–0.388	0.372	0.364	–0.253
P		<0.001	0.001	<0.001	0.051	<0.001	<0.001	<0.001	0.076
WHR									
R			0.522	0.509	–0.287	0.463	0.394	0.491	0.078
P			<0.001	<0.001	0.004	<0.001	<0.001	<0.001	0.595
SBP									
R				0.718	–0.224	0.269	0.310	0.234	–0.083
P				<0.001	0.025	0.007	0.002	0.020	0.566
DBP									
R					–0.154	0.231	0.227	0.197	–0.011
P					0.128	0.021	0.024	0.050	0.939
HDL-C									
R						–0.414	–0.191	–0.229	–0.167
P						<0.001	0.054	0.021	0.232
TG									
R							0.379	0.401	–0.130
P							<0.001	<0.001	0.353
FBG									
R								0.425	–0.163
P								<0.001	0.245
HOMA–IR									
R									–0.236
P									0.038

Body mass index, BMI; waist-to-hip ratio, WHR; systolic blood pressure, SBP; diastolic blood pressure, DBP; highdensity lipoprotein-cholesterol, HDL-C; low-density lipoprotein-cholesterol, LDL-C; triglyceride, TG; fasting blood glucose, FBG; Homeostatic Model Assessment-Insulin Resistance, HOMA-IR; correlation coefficient, R; probability, P.

**Table 3:** Results of logistic regression models predicting metabolic syndrome in psoriasis patients.

	OR (95% CI)	P-value
Adropin (ng/mL)	0.301 (0.103–0.878)	0.028*
BMI (kg/m2)	1.094 (0.863–1.387)	0.457
Waist circumference (cm)	0.986 (0.891–1.092)	0.792

*Significant at P < 0.05; body mass index, BMI.

## 4. Discussion

The results showed that serum adropin levels were low in psoriasis patients, and this reduction was more defined in patients who also had MetS. To our knowledge, this is the first study to identify an association between serum adropin levels and the risk of MetS in psoriasis patients.

Studies in recent times have shown that in psoriasis patients, the incidence of MetS and associated morbidities such as DM, obesity, HT, and dyslipidemia are increased. Additionally, endothelial functions are disrupted in psoriasis patients, and cardiovascular comorbidities are frequently encountered (15–17). The current study showed that HDL-C levels were lower in psoriasis patients without MetS compared to the healthy control group, while SBP and DBP levels were higher (Table 1). A study by Coban et al. identified higher DBP, FBG, and LDL-C levels in psoriasis cases without MetS compared to healthy controls (7). This finding was thought to be due to the disruption of metabolic parameters that starts before the development of MetS in psoriasis patients.

Adropin plays an important role in glucose and lipid homeostasis. It has a significant role in preventing the development of DM, dyslipidemia, impaired glucose tolerance, and insulin resistance. Increased adropin release in obese patients was observed to regulate liver fattening, glucose homeostasis, and dyslipidemia. Adropin additionally has a regulatory role in endothelial functions (9,18–20). A total rise of 50% in the fat tissue of adropin knockout mice was observed regardless of the nutritional intake and energy consumption. They were also shown to develop dyslipidemia and insulin resistance (10). The current study found a reduction in serum adropin levels in psoriasis patients; this reduction was more significant in psoriasis patients with MetS. A negative correlation was observed between adropin levels and TG and insulin resistance. Additionally, upon multivariate regression analysis, a decrease in adropin was identified as an independent risk factor for the presence of MetS in psoriasis patients.

A cohort study that included 4065 psoriasis patients showed a correlation between MetS components like obesity, hypertriglyceridemia, and disease severity (21). Another study of 127,706 psoriasis patients also identified this correlation (22). In the current study, no correlation was found between disease severity, including metabolic components, and adropin levels. This may have resulted from the relatively low number of patients recruited to the current study and also the presence of low number of moderate-severe psoriasis patients in the cohort. The limitation of this study was the lack of a second control group with metabolic syndrome.

In conclusion, the serum level of adropin in psoriasis patients was significantly lower compared to the healthy control group; additionally, this decrease was more prominent in psoriasis patients who also had MetS. Reduced serum adropin levels in psoriasis patients may be a factor responsible for metabolic disorders and development of MetS. Further studies may identify adropin as a biomarker for the development of treatments to reduce the cardiovascular morbidity in psoriasis patients.
